# The effects of early-life risperidone administration on forebrain neurotrophin expression during adulthood

**DOI:** 10.1186/1471-2105-13-S12-A19

**Published:** 2012-07-31

**Authors:** Matt Gannon, Rachel Stevens, Molly Griffith, Mark Bardgett

**Affiliations:** 1 Department of Psychological Science, Northern Kentucky University, Highland Heights, KY 41076, USA

## Background

The antipsychotic drug risperidone has become increasingly popular as a treatment for children with various behavioral disorders, including autism. However, little is known about the long-term effects of early-life risperidone treatment on the brain and behavior. The frontal cortex is one of the primary targets of risperidone action in the brain, so the purpose of this study was to determine if early-life risperidone treatment altered neurotrophin expression in the prefrontal cortex during adulthood.

## Materials and methods

Twenty-four rats (13 females, 11 males) received daily injections from postnatal days 14-42. Rats were divided into two treatment groups (1.0 and 3.0 mg/kg risperidone) and a vehicle control. Brain tissue was collected on postnatal day 62. Prefrontal cortical tissue from the left hemisphere was examined for neurotrophin expression through the use of a rat neurotrophin RT-PCR array kit (SABiosciences, Inc.). Sections of prefrontal cortex were also used for immunohistochemistry.

## Results

PCR results indicated an up-regulation of mRNA for the cytokine, Leukemia Inhibitory Factor (LIF), in the low and high dose treatment groups. Immunohistochemistry is being conducted to assess possible differences in LIF protein levels in the prefrontal cortex of the different treatment groups.

## Conclusion

This study indicates that early-life risperidone treatment has the potential to alter the expression of proteins involved in cell growth and differentiation with brain regions critical for cognitive control. The behavioral consequences of these cellular changes are an area that further research should address.

